# Supplementary figures and images for: Stress-dependent activation of PQM-1 orchestrates a second-wave proteostasis response for organismal survival
Source: bioRxiv. 2025 Mar 14:2025.03.11.642454. Preprint. [Version 1] doi: 10.1101/2025.03.11.642454 (PMC11952446; doi:10.1101/2025.03.11.642454)

Supplemental Figure 1

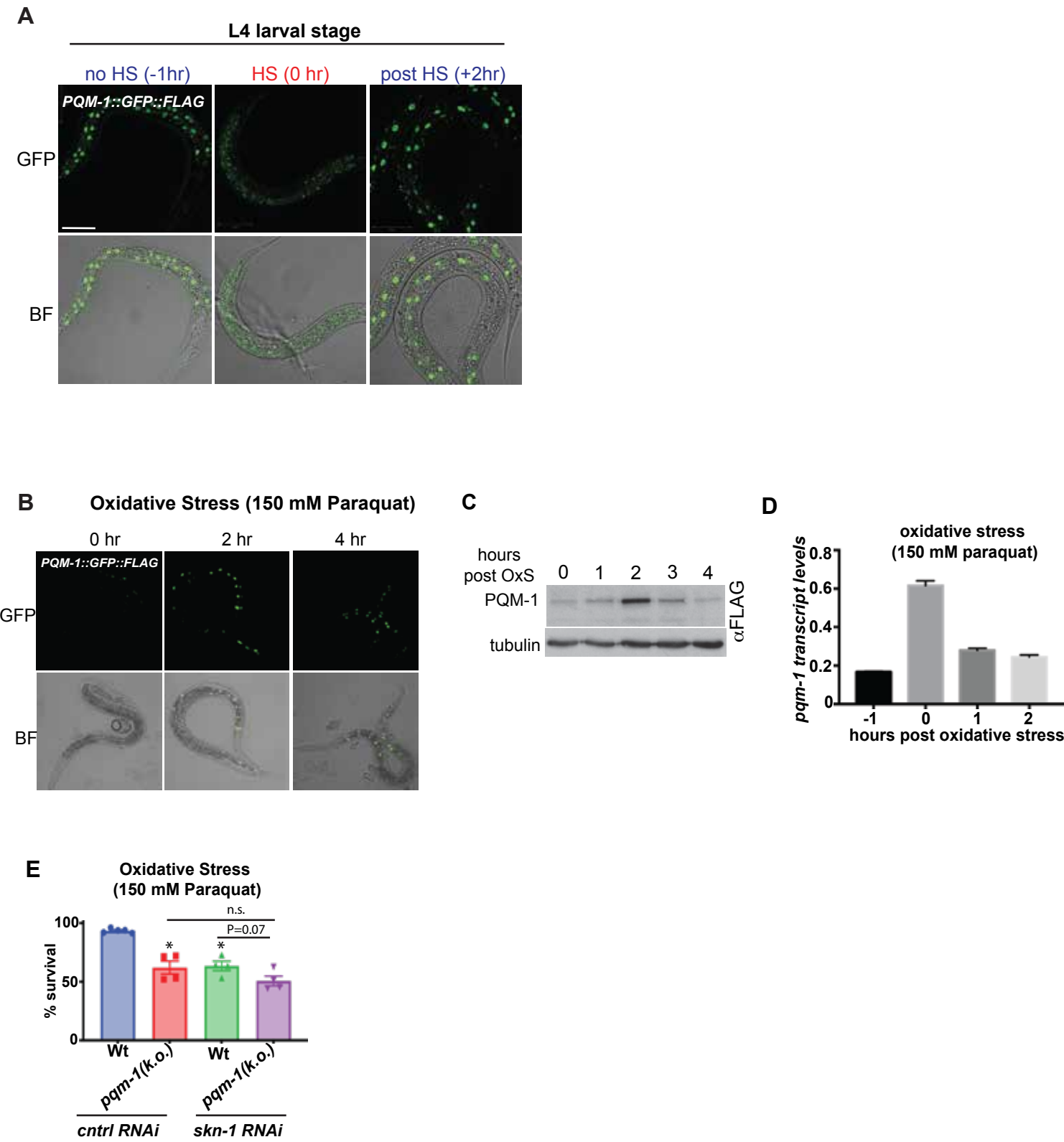

Supplemental Figure 2

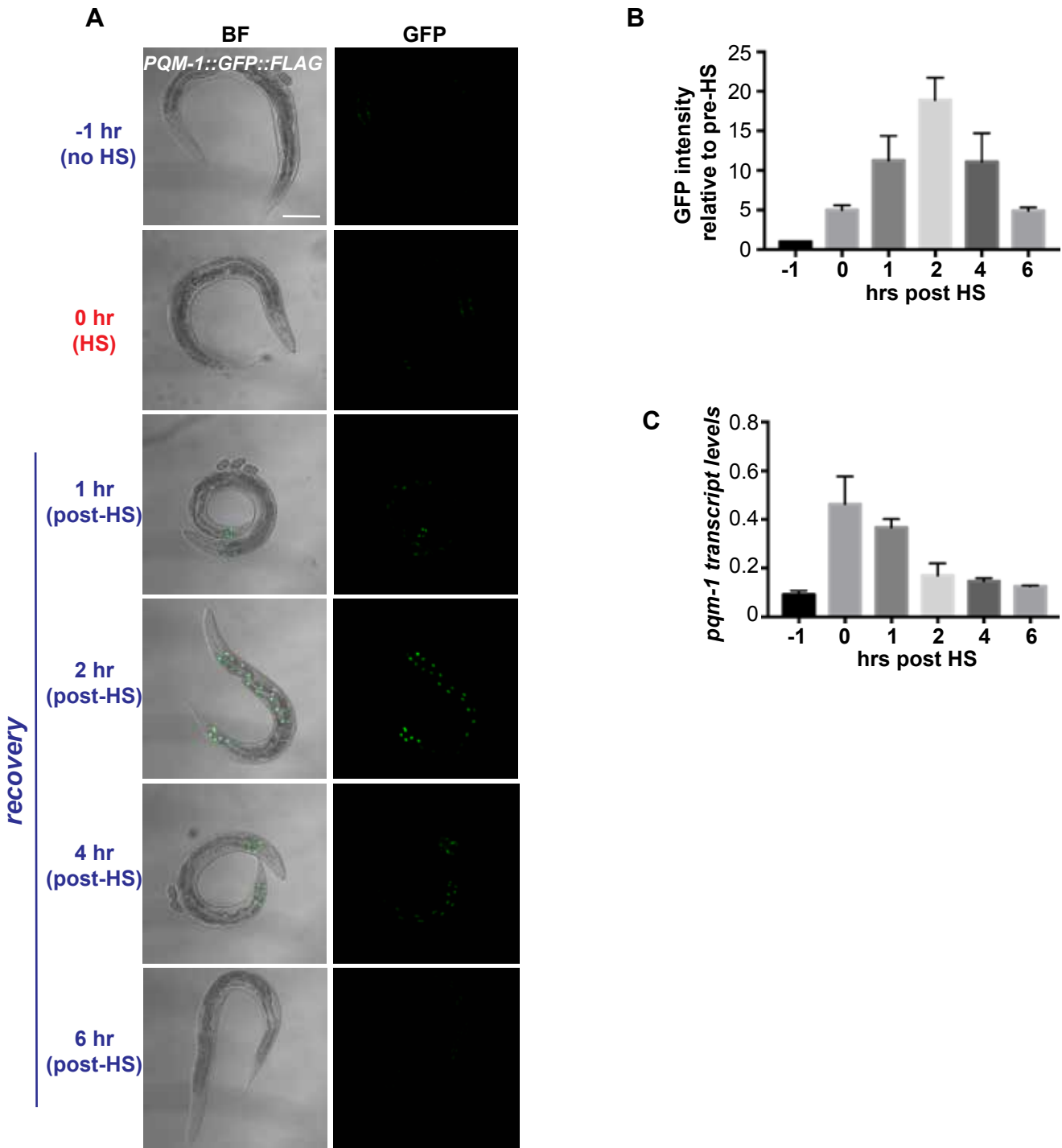

Supp Figure 3

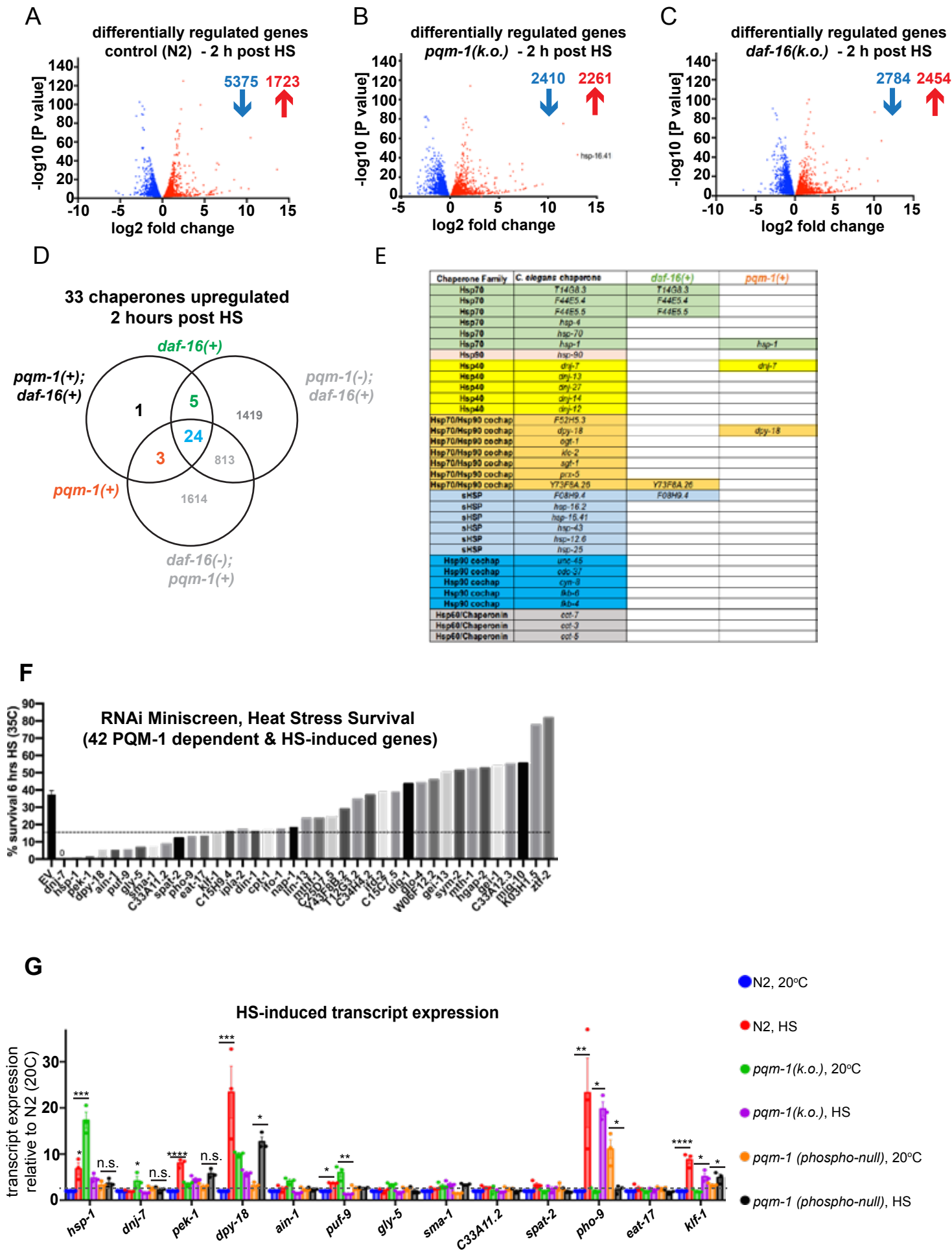

Supplement: Supplement 1 — Supplemental Figure 1. PQM-1 expression and nuclear localization dynamics during heat- and oxidative stress. (A) Confocal images of PQM-1::GFP expression in L4 larvae (strain OP201) before HS (−1 hr), immediately after a 1-hour HS at 35°C (0 hr) and after 2 hours of recovery post HS (+ 2hr). PQM-1::GFP is localized to intestinal nuclei in L4 stage larvae during normal growth conditions. (B) Confocal images of Day-1 adult PQM-1::GFP::FLAG expression after (0 hr, 2 hr and 4 hr) oxidative stress (150 mM paraquat). (C) Western blot analysis of PQM-1::GFP::FLAG expression immediately after (0 hr) and 1 – 4 hrs post oxidative stress, using an anti-FLAG antibody and tubulin as a loading control. (D) pqm-1 transcript levels before (−1 hr) and 0, 1, 2, 4, 6 hours after a 1-hr exposure to oxidative stress (150 mM paraquat). (E) Survival to oxidative stress of Day 1 adults grown on control or skn-1 RNAi and treated with 150 mM Paraquat for 6 hours. n = 20; three biological replicates. Bar graph represent SEM, *P < 0.05; n.s. = not significant. Supplemental Figure 2. PQM-1 nuclear localization and transcript expression before and after HS. (A) Confocal images of PQM-1::GFP expression in Day 1 adult nematodes before HS (− 1hr), immediately after a 1-hour 35C HS (0 hr) and 1, 2, 4 and 6-hours post HS. Scale bar = 50 μm. (B) Nuclear PQM-1::GFP fluorescence intensity before and after HS in Day-1 adults. (C) pqm-1 transcript levels before (−1 hr) and 0, 1, 2, 4, 6 hours after HS. Supplemental Figure 3. RNA-Seq analysis of pqm-1 dependent gene expression in heat-shocked Day 1 adults. (A) RNA-Seq scatterplot showing log2 fold-changed expression levels of differentially expressed genes (P value < 0.05) 2 hours after HS in control (N2) worms (B) Scatterplot showing log2 fold-changed expression levels of differentially expressed genes (P-Value < 0.05) in pqm-1(ko) mutants 2 hours post HS. (C) Scatterplot of log2 fold-changed gene expression levels (P < 0.05) in daf-16 mutants 2 hours pos [file media-1.pdf]
